# Emigration, remittances, and the subjective well-being of those staying behind

**DOI:** 10.1007/s00148-018-0718-8

**Published:** 2018-08-09

**Authors:** Artjoms Ivlevs, Milena Nikolova, Carol Graham

**Affiliations:** 1 Bristol Business School, University of the West of England, Bristol, BS16 1QY UK; 2 IZA, Bonn, Germany; 3 Faculty of Economics and Business, Global Economics and Management, University of Groningen, Nettelbosje 2, 9747AE Groningen, The Netherlands; 4 The Brookings Institution, Washington, DC USA

**Keywords:** Migration, Remittances, Depression, Stress, Cantril ladder of life, Happiness, Gallup World Poll, F22, F24, I31, O15

## Abstract

We offer the first global perspective on the well-being consequences of emigration for those staying behind using several subjective well-being measures (evaluations of best possible life, positive affect, stress, and depression). Using the Gallup World Poll data for 114 countries during 2009–2011, we find that having family members abroad is associated with greater evaluative well-being and positive affect, and receiving remittances is linked with further increases in evaluative well-being, especially in poorer contexts—both across and within countries. We also document that having household members abroad is linked with increased stress and depression, which are not offset by remittances. The out-migration of family members appears less traumatic in countries where migration is more common, indicating that people in such contexts might be able to cope better with separation. Overall, subjective well-being measures, which reflect both material and non-material aspects of life, furnish additional insights and a well-rounded picture of the consequences of emigration on migrant family members staying behind relative to standard outcomes employed in the literature, such as the left-behind’s consumption, income, or labor market outcomes.

## Introduction

Owing to high migration costs, strict migration policies, and uncertain conditions at the destination, international migrants often leave family members in the countries of origin (Démurger 2015). The literature shows that migration and remittances can affect various socio-economic outcomes among those left behind, such as poverty and income (Adams 2011; Gibson et al. 2011), education (Antman 2012; Cortes 2015; Kroeger and Anderson 2014; Yang 2008), and health (Antman 2010; Böhme et al. 2015; Gibson et al. 2011; Kroeger and Anderson, 2014). Migrants can also change norms, attitudes, and behaviors back home. Examples of such non-monetary, or social (Levitt 1998), remittances include the effects of emigration on political participation (Chauvet and Mercier 2014), corruption behavior (Ivlevs and King 2017), fertility (Beine et al. 2013), and civic engagement (Nikolova et al. 2017). While not all studies point to superior socio-economic, behavioral, and health outcomes for those left behind, migration and remittances have been increasingly recognized as important development tools for the origin countries (Skeldon 2008; UNDP 2009).

There has recently been increasing academic and policy interest in the subjective well-being consequences of migration for household members staying behind in the origin country. The literature has mainly focused on children, their caregivers, and the elderly, with the results varying depending on the nature of migration (internal or international), who is left behind (e.g., children vs. parents), the outcome measure and the analysis country or countries. For example, Dreby (2015) and Wu et al. (2015) document greater feelings of resentment and depression among children of emigrant parents in Mexico and China, while Vanore et al. (2015) find that parental out-migration is unassociated with children’s emotional well-being (an index based on information on the feelings of worry, unhappiness, nervousness, and fear) as well as conduct problems in Moldova. A study on Ghana, Angola, and Nigeria (Mazzucato et al. 2015) reveals that changing caregivers due to the out-migration of family members negatively affects children’s psychological well-being (a composite measure of psychological distress derived from the Strength and Difficulties Questionnaire (Goodman 1997)); in addition, the type of migration (internal or international) and which parent migrates matters in some country contexts but not others. Fathers’ migration is associated with children’s conduct problems in Thailand and Moldova (E. Graham and Jordan 2011; Vanore et al. 2015) but not in China, where father-only migration is linked with a lower likelihood of problem behaviors among children (Wen et al. 2015).

Looking at the mental health of migrant children caregivers in South-East Asia, E. Graham et al. (2015) find that mothers whose partners have migrated are more likely to suffer from poor mental health (measured using an index based on self-reported emotional distress, including nervousness, difficulty in making decisions, suicidal thoughts, tiredness, headaches, and poor appetite) than mothers from non-migrant households. Similarly, Nobles et al. (2015) document increased sadness, crying, and difficulty sleeping among the stay-behind mothers in Mexico. The mental health of the elderly parents was found to deteriorate after the migration of children in China and South Africa (Marchetti-Mercer 2012; Scheffel and Zhang 2015; Xie et al. 2014). The evidence for Thailand is more mixed, with Adhikari et al. (2011) reporting a negative association and Abas et al. (2009) finding the opposite. Providing causal estimates is a common challenge (Démurger 2015), and the few studies explicitly addressing causality (Böhme et al. 2015; Gibson et al. 2011; Waidler et al. 2016) find that emigration has no effect on the mental health (captured by various indicators, including an index of feeling happy, peaceful, tense, blue and downhearted, and feeling depressed) of the elderly staying behind in Moldova and Tonga.^1^

An emerging literature has considered the well-being consequences of receiving migrant remittances from abroad (which we define as transfers of money and goods made by migrants to the family members back home; henceforth, remittances).^2^ For example, remittance receipt is positively associated with life satisfaction in Latin America, possibly through increasing financial security (Cárdenas et al. 2009). Borraz et al. (2010) find that migrant and non-migrant households experience similar happiness levels, arguing that remittances compensate migrant households for the pain of separation and the disruption of family life. Gartaula et al. (2012) find that Nepalese women in remittance-receiving households experience improvements in objective well-being (economic situation, access to food and water, child education, etc.) but not necessarily subjective well-being (feeling separated from partner, feeling overburdened with work, problems with disciplining children, stricter control from parents-in-law). Investigating rural-migrant migration in China, Akay et al. (2016) document that remittance income is positively associated with mental health (as measured by the GHQ-12 questionnaire) among the left behinds of rural-to-urban migrants but having one or more migrant workers in the family is negatively associated with mental health.

With some exceptions (Cárdenas et al. 2009; E. Graham and Jordan 2011; E. Graham et al. 2015; Mazzucato et al. 2015), the existing evidence has focused on data from a single—and predominantly low or lower-middle-income—origin country, leaving the heterogeneity in the relationship between emigration and the well-being of those staying behind unexplored across diverse countries of origin. This paper fills this knowledge gap by studying emigration’s well-being consequences in a wide range of origin countries, including high-income countries, and using several subjective well-being dimensions, which has not been previously done in the literature. In particular, the term “subjective well-being” refers to both hedonic (i.e., *affective*) and cognitive (i.e., *evaluative*) dimensions of well-being. Positive hedonic well-being encompasses positive feelings *at a particular point in time* such as joy and happiness. Negative hedonic well-being includes experiences of stress, anger, sadness or worry at a particular point in time.^3^ In contrast, evaluative well-being is an overall cognitive reflective assessment of the respondent’s life as a whole. Evaluative well-being usually reflects people’s capabilities, means, and long-term opportunities (Graham and Nikolova, 2015). This dimension is typically measured using general life satisfaction questions or the Cantril ladder of life question, whereby respondents rate their current life on an 11-point scale, where 0 represents their worst possible life and 10 corresponds to the best possible life that they can imagine for themselves.^4^ Assessing to what extent one’s life is the best possible one can imagine for her/himself requires a thorough evaluation of past and present life circumstances. By contrast, hedonic experiences indicate emotions and moods triggered by pleasant and unpleasant daily experiences such as commuting, minor health conditions such as having a cold, spending time with family or friends, or reading a funny book. As explained in Section 2.2, in this paper we utilize four subjective well-being outcome variables. First, our *evaluative well-being* proxy is based on the Cantril ladder of life question (best possible life (BPL)). The rest of our dependent variables capture hedonic well-being dimensions, which reflect short-term positive and negative moods related to daily lives and activities.

Relying on Gallup World Poll data and evaluative and hedonic well-being measures, we ask the following questions: what is the relationship between the out-migration of family members and different subjective well-being dimensions of household members staying behind? Do income levels—both between and within countries—affect this relationship? What is the role played by remittances?

Finding answers to these questions is important from a policy perspective for the following reasons. First, subjective well-being relates to the notion that how people experience a set of objective circumstances may be just as important as those circumstances themselves and that individuals are the best judges of how their lives are going (OECD 2011). By reflecting both objective and perceived circumstances, subjective well-being is an integrated representation of individual welfare. Unsurprisingly, governments around the world are increasingly complementing objective welfare metrics with subjective well-being outcomes such as life satisfaction and happiness to assess individual welfare and societal progress and guide policy-making (O'Donnell 2013; OECD 2013; Office for National Statistics 2013). In the context of our study, subjective measures allow us to draw a more rounded picture of the effects of emigration on migrant family members staying behind than by simply looking at the left-behind’s consumption, income, or labor market responses. Second, subjective well-being is important to policy-makers as it has a number of objective benefits. For example, higher subjective well-being levels are linked with better physical health and longevity, given that happier people live longer, have better cardiovascular and immune systems, recover quicker from illnesses, exercise more, have better eating habits, and are less likely to adopt risky health behaviors (De Neve et al. 2013; Diener and Chan 2011; Howell et al. 2007; Sabatini 2014). Happier people also have greater social skills and are more productive, creative and motivated in the workplace (De Neve et al. 2013; Oswald et al. 2015).

We argue that the emigration of household members can be linked with multiple—often conflicting—subjective well-being states among those staying behind. For example, the pain of separation from family members could provoke increased stress and depression (i.e., negative hedonic components of subjective well-being), possibly more so in countries where emigration is less common and people have not developed mechanisms to deal with separation. The out-migration of a family member who was helping through market or household production at home could also lead to family disruptions and thus lower subjective well-being (Borraz et al. 2010). At the same time, knowing that family members have more opportunities and realize their potential though emigration could result in greater life satisfaction and more positive life evaluations (i.e., cognitive components of subjective well-being). In other words, the left behind family member could have altruistic feelings towards the migrant, who may be leading a better life abroad. Many migrants send home money, which could compensate for any negative separation effects through increasing income and opportunities, as well as reducing vulnerabilities, and thus boosting subjective well-being. This conjecture is supported by the New Economics of Labor Migration (NELM) framework, according to which households send migrants abroad with a prospect of receiving remittances that would subsequently be used to invest in new activities or insure against risks (Taylor 1999). One could thus expect a positive link between remittance receipt and well-being (through increased capabilities and greater security), especially in poorer countries, where credit and insurance markets perform less well, as well as among poorer households, who may face greater obstacles in securing credit and insurance through formal channels.

To furnish a global perspective of the relationship between emigration and the subjective well-being of household members staying behind, we use data from the Gallup World Poll (GWP), which include several subjective well-being questions and information on whether the respondent has household members abroad who left in the past five years. Our analysis sample spans 114 countries, allowing us to uncover both the common trends in a set of varied countries and differences across country groups.

Our study contributes to the scholarly dialog and the burgeoning literature on the well-being of those staying behind by providing a global perspective, i.e., exploring the subjective well-being consequences of emigration in a wide range of origin countries.^5^ In this sense, this study is the first to furnish evidence on the well-being benefits and costs of emigration in high-income countries. Second, we contribute to the broader literature exploring the links between migration and subjective well-being (typically measured with life satisfaction and happiness).^6^ While existing studies have examined the relationship between immigration and the subjective well-being of migrant-receiving populations (Akay et al. 2014a, 2017a, Betz and Simpson, 2013; Ivlevs and Veliziotis 2018; Longhi 2014), the impact of home-country conditions on migrants’ happiness abroad (Akay et al. 2017b), migration’s consequences for migrants’ subjective well-being (Nikolova and Graham 2015), as well as the effects of subjective well-being on the decision to emigrate (Cai et al. 2014; Graham and Markowitz 2011; Ivlevs 2015; Otrachshenko and Popova 2014), we add to this literature by looking at the effects of emigration on the well-being of those staying behind in the countries of origin.

## Method

### Data

The data in this paper are from the GWP, an annual global survey conducted since 2005/6 in about 160 countries worldwide, representing more than 99% of the world’s civilian non-institutionalized population aged 15 and older. Polling approximately 1000 respondents in each country (with one respondent per household), Gallup asks a core set of questions using face-to-face or phone interviews (where telephone coverage is more than 80%). With few exceptions (e.g., when interview staff’s safety is compromised), all samples are probability-based and nationally representative.^7^ One key advantage of the GWP for the purposes of our analysis is that it collects subjective well-being data along several dimensions and according to the OECD Guidelines (2013).

Since 2009, Gallup has provided household income and employment information, and thus we use 2009 as the starting point for this analysis. Our analysis sample is also based on all available countries and years since 2009 with valid information on whether: (i) the members of the respondent’s household have moved abroad permanently or temporarily in the past 5 years and are still there; and (ii) the respondent’s household has received help in the form of money or goods from abroad in the past 1 year. While the first variable informs whether family members left in the past 5 years, we do not have information on the exact duration of the migration episode; furthermore, there is no information on the minimum amount of time that an individual should spend abroad to be considered a migrant. Other limitations of the emigration of family members variable—which we acknowledge but cannot correct—include the lack information on whether the migrant is abroad permanently or temporarily (e.g., circular migrant, temporary migrant, studying abroad) and what the exact familial relationship of the emigrant to the respondent is.

Our sample (*N* = 144,003) comprises 114 countries and spans the period 2009–2011 (some countries appear in all 3 years), with the majority (78%) of observations coming from 2009 (countries are listed in Table 12 in the Appendix).^8^ In Section 3.2, we provide additional specifications for 2009 only, for the Western Balkan countries (which are the only country group appearing in all 3 years), and offering weighted regressions (using the inverse of the number of years in the regressions as a weight).

### Variables

#### Dependent variables

As subjective well-being is a multidimensional construct (OECD, 2013), we use four individual-level outcome variables, which has not been previously done in the literature on the well-being consequences of emigration for the left behind. Evaluative well-being is based on a question on the best possible life (BPL), whereby respondents are asked to evaluate their current life on a ladder from 0 (worst possible) to 10 (best possible that life they can imagine for themselves). In contrast to this evaluative subjective well-being dimension, the rest of our dependent variables capture hedonic well-being dimensions, which reflect short-term positive and negative moods related to daily lives and activities. Specifically, using principal component analysis, we construct a positive affect index, which is the first principal component of three binary variables capturing the experience of joy, happiness, and smiling the day before the interview. To be consistent with the evaluative well-being (BPL) measure, we re-scale the index—which captures positive hedonic well-being—to range from 0 to 10. Next, we include two separate binary variables capturing the experience of stress and depression. We refrain from constructing a negative affect index from these variables because—in contrast to positive ones—negative hedonic well-being dimensions tend to be more differentiated and multidimensional (Stone and Mackie, 2014). In addition, we are particularly interested in how depression experiences, which are a marker of mental health, relate to the emigration of household members. We are confident in performing cross-country analyses of these subjective well-being measures, as psychological and brain-scan research indicates that they are consistent across time and space (see, e.g., C. Graham, 2009) and the effect of cultural biases on answering subjective well-being questions is limited (Exton et al. 2015).

#### Independent variables

We include two focal independent variables: (i) whether the members of the respondent’s household have moved abroad permanently or temporarily in the past 5 years and are still there; and (ii) whether the respondent’s household has received help in the form of money or goods from abroad in the past year. When included in the estimations jointly, the coefficient estimate on remittances will capture the monetary consequences of migration for the well-being of those left-behind such as the additional well-being received through the increase in disposable income,^9^ while the coefficient estimate on the having family abroad variable reflects the residual migration effect, which, among other things, captures the psychological consequences (both positive and negative) of the out-migration of family members for those left behind at the origin.

#### Control variables

Our control variables comprise standard individual and household socio-demographic characteristics, namely, the respondent’s age, gender, education, marital status, children in the household, urban or rural location, household size, employment status, and religiosity (whether religion is important in the respondent’s life); all variable definitions are provided in Table 11. Importantly, we also control for within-country household income quintiles, and as such, any conditional correlations that we identify between our key independent variables and subjective well-being are above and beyond the influence of household income per se. We also include three self-reported health variables: experiencing physical pain, health satisfaction, and whether the respondent reported a health problem. We do so to separate subjective well-being from physical health as much as possible, as health conditions may affect subjective well-being (C. Graham et al. 2011). In addition, health conditions may affect the probability of staying behind, which is why we need to control for them in the regression.^10^

To avoid bias from dropping observations due to missing data, we create an additional category for missing observations for all variables included in the analyses. Regressions using only non-missing observations are consistent with our main findings and are reported in Table 17 in the Appendix.

### Estimation strategy

In separate regressions, we estimate the association between each of the four subjective well-being outcomes (evaluative well-being measured as the respondent’s assessment of the best possible life (BPL)), positive affect, stress, depression) and the out-migration of a household member, using an ordinary least squares (OLS) estimator. While the evaluative well-being (BPL) variable is ordinal and technically we need an ordinal logit or an ordinal probit estimator, Ferrer-i-Carbonell and Frijters (2004) show that the results do not differ when OLS is used with ordinal subjective well-being data. OLS estimations are moreover easier to interpret. For consistency, we also estimated with OLS the models explaining stress and depression, where the dependent variable is binary.^11^

The subjective well-being outcome *S* of individual *i* in time period *t* living in country *c* is

1 $$ {S}_{\mathrm{itc}}=\alpha +{\beta}_1{M}_{\mathrm{itc}}+{\beta}_2{R}_{\mathrm{itc}}+X{\prime}_{\mathrm{itc}}\gamma +{\pi}_c+{\tau}_t+{u}_{\mathrm{itc},} $$

where *M* is a binary indicator for having a household member abroad who has emigrated in the past 5 years, *R* is a binary indicator for whether the respondent lives in a remittance-receiving household, *X* is a vector of individual- and household-level characteristics, *π*_*c*_ are country dummies, τ_t_ are year dummies, and *u*_itc_ is the stochastic error term. Simultaneously including both focal independent variables in the same regression allows us to discern the contribution of the financial boost (if any) from remittances for subjective well-being above and beyond that of having family abroad.

At the outset, we note that our results should be interpreted as *conditional correlations* rather than *causal effects*. The main concern relates to the fact that the emigration does not occur at random. Traits such as openness, risk aversion, motivation, and ability could affect both well-being and the selection of individuals into migration both within and across households. The lack of panel data—whereby the same migrants and their family are observed over time and where appropriate, across international borders—does not allow us to control for such unobserved, time-invariant characteristics that simultaneously influence subjective well-being and emigration.^12^Another source of endogeneity is reverse causality, as it is conceivable that the deteriorating subjective well-being of household members is part of the migration decision. For example, if the subjective well-being of parents is ex ante poor, then the likelihood that their children emigrate is lower (Démurger, 2015). It is also possible that unhappy family members make it more likely that other members choose to move away (Borraz et al. 2010). Nevertheless, additional estimates in Table 18 of the Appendix demonstrate that while some subjective well-being dimensions are determinants of having a migrant family member abroad and, to some extent, receiving remittances, they only predict at most 1% of the probability of having a family member or receiving remittances. Depression and stress feelings are not associated with remittances, moreover (models (6) and (8) in Table 18). Thus, while reverse causality may be possible, it is unlikely that it is driving all of our findings.

Correcting reverse causality and selection bias is usually achieved using instrumental variables (Böhme et al. 2015; Waidler et al. 2016), natural experiments (Gibson et al. 2011), or selection-correction procedures and matching (Borraz et al. 2010). Nonetheless, finding convincing instruments that are only correlated with the migration decision but not subjective well-being is challenging. Böhme et al. (2015) study the consequences of children’s out-migration on the health of elderly left behind parents in Moldova. The authors demonstrate that selection biases simple OLS results downwards, implying that when the selection of individuals from poor households with a priori sickly parents is taken into account using instrumental variables approach, the true positive consequences of emigration for the health of the elderly left behind are even stronger. Waidler et al. (2016) reach the opposite conclusion, again using a similar sample for Moldovan elderly parents and an instrumental variable estimation. Finally, as noted, using an experiment involving a migration lottery allowing Tongans to emigrate to New Zealand, Gibson et al. (2011) do not find much evidence that self-selection at the individual level biases the results. Additionally, matching methods such as those used in Borraz et al. (2010) assume that the selection into migration is based on observables, which is also methodologically problematic. It is thus difficult to know whether or not selection may be plaguing our results. Based on the experimental evidence of Gibson et al. (2011) and our own estimates using regressions applied after entropy balancing, selection should not be the main driver of our findings. Yet, we do not have experimental findings against which we can benchmark our estimates. While we acknowledge possible endogeneity issues and do our best to mitigate them, our goal is to offer the first global assessments of the patterns in the relationship between emigration and the well-being of those left behind, while leaving causal explorations to further research. With these caveats in mind, we apply additional caution when interpreting our results. Nevertheless, we show that our finding survive several sensitivity tests, which suggests that while selection may be a problem, it is not the primary driver behind our results.

## Results

### Baseline results

Table 1 reports our main estimation results. Holding constant the other control variables, both remittances receipt and having family members abroad are positively and statistically significantly associated with life evaluations (BPL) (model (1)). In other words, remittances have a positive and significant association with BPL beyond the influence of *relatives abroad*. Specifically, remittance receipt corresponds to a 0.11 point increase in life evaluations, which, evaluated at the sample mean of 5.495 (see Table 13 in the Appendix for summary statistics), is linked with a 2% increase in life evaluations (BPL), a result that is statistically significant but relatively small in terms of magnitude. This result is likely due to the increase in material living standards, or a “signaling effect” (Akay et al. 2016), which could also allow for the expanded capabilities and means that remittances bring. The signaling effect could reflect the different social status remittance-receiving families could have in the community.

**Table 1:** Emigration of family members, remittances, and subjective well-being of those staying behind, full sample, ordinary least squares results, 2009–2011

	BPL (0/10)	Positive affect (0/10)	Stress (0/1)	Depressed (0/1)
	(1)	(2)	(3)	(4)
Relatives abroad	0.082***	0.104***	0.010***	0.008***
	(0.016)	(0.029)	(0.004)	(0.003)
Remittances	0.105***	0.081**	− 0.003	0.002
	(0.020)	(0.037)	(0.005)	(0.004)
Ages 36–60	− 0.225***	− 0.465***	− 0.006**	0.015***
	(0.012)	(0.022)	(0.003)	(0.002)
Over 60	− 0.172***	− 0.601***	− 0.099***	− 0.014***
	(0.019)	(0.033)	(0.004)	(0.003)
Female	0.108***	0.097***	0.020***	0.006***
	(0.010)	(0.019)	(0.002)	(0.002)
Married/living with partner	0.022**	0.138***	0.001	− 0.014***
	(0.011)	(0.021)	(0.003)	(0.002)
Children in household	− 0.100***	−0.050**	0.018***	0.006***
	(0.014)	(0.025)	(0.003)	(0.002)
Household size	0.062***	0.087***	0.001	−0.004***
	(0.005)	(0.009)	(0.001)	(0.001)
Household size^2^/100	− 0.239***	− 0.318***	0.000	0.020***
	(0.029)	(0.051)	(0.006)	(0.005)
Second income quintile	0.253***	0.181***	− 0.013***	− 0.021***
	(0.018)	(0.033)	(0.004)	(0.003)
Third income quintile	0.498***	0.339***	− 0.021***	−0.027***
	(0.018)	(0.033)	(0.004)	(0.003)
Fourth income quintile	0.665***	0.501***	− 0.028***	− 0.041***
	(0.018)	(0.032)	(0.004)	(0.003)
Richest 20%	0.972***	0.761***	− 0.037***	− 0.051***
	(0.018)	(0.033)	(0.004)	(0.003)
Secondary education	0.305***	0.203***	0.011***	− 0.015***
	(0.013)	(0.024)	(0.003)	(0.002)
Education missing	0.331***	0.044	− 0.005	− 0.030***
	(0.064)	(0.103)	(0.015)	(0.011)
Unemployed	− 0.498***	− 0.359***	0.005	0.049***
	(0.028)	(0.048)	(0.006)	(0.005)
Out of the labor force	0.085***	0.141***	− 0.060***	−0.001
	(0.012)	(0.022)	(0.003)	(0.002)
Pain yesterday	− 0.239***	−1.370***	0.187***	0.141***
	(0.013)	(0.025)	(0.003)	(0.003)
Dissatisfied with health	− 0.766***	−1.334***	0.080***	0.080***
	(0.015)	(0.029)	(0.004)	(0.003)
Has a health problem	− 0.136***	− 0.183***	0.023***	0.038***
	(0.014)	(0.026)	(0.003)	(0.003)
Religion important	0.073***	0.396***	− 0.010***	0.001
	(0.014)	(0.026)	(0.003)	(0.002)
Large city	0.122***	0.039*	0.025***	0.013***
	(0.012)	(0.021)	(0.003)	(0.002)
Country and survey wave dummies	Yes	Yes	Yes	Yes
Observations	142,468	121,607	126,803	126,680
Adjusted *R*^2^	0.283	0.199	0.109	0.104

Source: Authors’ estimation based on Gallup World Poll data

Notes: Robust standard errors in parentheses. ***p < 0.01, **p < 0.05, *p < 0.1. The omitted categories are ages 15–35; completed primary education; married or living with partner; poorest 20%; no children in the household; small city/village; employed (full- or part-time, or self-employed); religion unimportant; no pain yesterday; satisfied with personal health; no health problem. Dummy variables for missing observations for each variable included but not reported. See Table 11 and Table 12 in the Appendix for variable definitions and the list of countries included in each survey wave

There is an additional residual migration effect, as captured by the relatives abroad variable, which is about the same size of that of remittances. This residual migration effect could reflect the subjective well-being derived from aspiration fulfillment at the household level. Put differently, if emigration of household members is a household decision, then families left behind at the origin may derive satisfaction from the fact that migrants realize their potential abroad. Having a migrant abroad could also increase the opportunity for the respondent to move abroad in the future, hence raising the evaluation of one’s best possible life (BPL).

Similarly to the results in model (1), those in model (2) in Table 1 suggest that both remittance-receipt and the residual migration effects are associated with higher levels of positive affect among those staying behind. Evaluated at the sample mean (7.205), the estimated coefficient for remittance-receipt in model (2) is associated with a 1.4% increase in the average positive affect score, which is also relatively small. The associated residual migration effect (i.e., the migration effect above and beyond the effect of income received through remittances) is also positive, statistically significant, and similar in magnitude to the remittance variable. Thus, the out-migration of family members seems to positively influence both life evaluations and positive emotions through both the income channel (remittances) and the residual psychological channel (having relatives abroad).

Despite being positively linked with evaluative and hedonic well-being, remittance receipt is a statistically insignificant predictor of stress and depression (models (3) and (4)), while the residual migration effect (relatives abroad) is positive and statistically significant. The positive residual migration effect likely reflects the worsened daily experiences related to the pain of separation, and the insignificant coefficient of remittances variables suggests the higher status and greater capabilities associated with receiving remittances do not reduce stress and depression experiences in respondents’ daily lives. Thus, while remittances “buy happiness” (i.e., contribute to BPL and positive affect above and beyond the relatives abroad variable), they do not relieve the pain of separation. Importantly, the conditional difference in the average stress scores between migrant and non-migrant households (0.010) in model (3) represents 3.9% of the average sample stress level (0.259). Having a household member abroad is linked with a 0.008 percentage point increase in the probability of reporting depression, which represents an increase of 6.5% relative to the average incidence of depression (0.124).

We also briefly comment on the estimated coefficients of the control variables in Table 1, most of which corroborate previous findings in the literature. People in the middle of the age distribution (ages 36–60) report lower BPL levels (on a scale of 0–10) as well as higher levels of depression compared to the young, whereas the elderly report the lowest levels of positive affect and the lowest levels of stress among all age groups. Women have on average higher life evaluation (BPL) and positive hedonic scores than men, suggesting, colloquially, that “women are happier than men,” although they are also more likely to report higher levels of stress and depression. Married respondents have higher levels of BPL, positive affect, and lower levels of depression, while having children is associated with lower levels of all types of subjective well-being. The statistically significant coefficients of the household size variable and its square imply a quadratic relationship between household size and evaluative and positive hedonic well-being, whereby a greater household size is associated with higher evaluative well-being (BPL) and positive affect, peaking when the household size reaches 14–16 and decreasing thereafter. Household size is negatively associated with depression experiences, although the relationship becomes positive after household size reaches 12. Being in a higher within-country income quintile is positively associated with both evaluative and hedonic well-being and is negatively linked to stress and depression. Holding constant the other included covariates, more educated people report higher evaluative well-being (BPL) and positive affect levels, higher stress levels, and lower depression levels. Relative to employed respondents, the unemployed report lower—and those out of labor force higher—levels of BPL and positive affect. Moreover, the unemployed are also more likely to experience depression and those out of labor force are less likely to report stress. As expected, inferior health (physical pain, health dissatisfaction, and health problems) is strongly associated with lower levels of evaluative and hedonic well-being, as well as increased stress and depression. Respondents for whom religion is important have better subjective well-being outcomes in all dimensions except depression, where the coefficient estimate is insignificant. Finally, respondents living in large cities (as opposed to small towns and villages) have higher levels of evaluative well-being (BPL) and positive affect, as well as stress, and depression.

### The role of income

The next set of analyses tests whether income levels—both across and within countries—affect the relationship between the out-migration of family members, receiving remittances and subjective well-being. First, Table 2 shows the results for the four country groups based on the World Bank’s per capita country income classification (see Table 12 in the Appendix for classifications). Panel A’s main takeaway is that as country income per capita decreases, the magnitude of the association between receiving remittances and evaluative well-being becomes stronger and peaks for lower-middle-income countries. For low-income countries, the BPL premium from migration is entirely driven by remittances. This is a novel finding, which was previously undocumented in the literature and implies that remittances play a *greater* role in enhancing evaluative well-being in *poorer* rather than in *richer* countries. A possible explanation—consistent with the NELM predictions—is that remittances expand the means and capabilities of the recipients and add to the feeling of financial security in poorer countries, where poverty is widespread, social welfare systems are weak, and credit and insurance markets are typically dysfunctional. As the marginal utility of income is higher and material means are more important for life evaluations in poorer rather than in richer countries, remittances are associated with higher well-being in the former. Meanwhile, remittances play no role for BPL in high-income countries, but having a migrant does, suggesting the different nature of the migration streams from these countries. Specifically, migrants from high-income countries emigrate to seek better opportunities abroad and family members back home feel reassured that their relatives are expanding their capabilities abroad.

**Table 2:** Emigration of family members, remittances, and psychological well-being of those staying behind, by country income group, 2009–2011

	High-income countries	Upper-middle income countries	Lower-middle income countries	Low-income countries
	Panel A: best possible life (0/10)			
Relatives abroad (1 = yes)	0.088**	0.117***	0.061**	0.053
	(0.037)	(0.031)	(0.027)	(0.036)
Remittances (1 = yes)	− 0.074	0.068*	0.183***	0.109***
	(0.075)	(0.039)	(0.033)	(0.038)
Observations	28,458	46,325	46,733	20,952
Adjusted *R*^2^	0.258	0.257	0.192	0.160
	Panel B: positive affect index (0/10)			
Relatives abroad (1 = yes)	0.047	0.090*	0.148***	0.063
	(0.078)	(0.051)	(0.047)	(0.075)
Remittances (1 = yes)	0.080	− 0.025	0.141**	0.119
	(0.169)	(0.065)	(0.056)	(0.085)
Observations	23,727	42,976	36,220	18,684
Adjusted *R*^2^	0.161	0.226	0.199	0.210
	Panel C: stress yesterday (0/1)			
Relatives abroad (1 = yes)	0.019*	0.007	0.011*	0.004
	(0.011)	(0.007)	(0.006)	(0.008)
Remittances (1 = yes)	0.019	− 0.008	0.001	− 0.011
	(0.022)	(0.008)	(0.008)	(0.009)
Observations	24,828	45,143	37,887	18,945
Adjusted *R*^2^	0.086	0.092	0.131	0.122
	Panel D: depressed yesterday (0/1)			
Relatives abroad (1 = yes)	0.002	0.007	0.015***	0.004
	(0.007)	(0.005)	(0.005)	(0.008)
Remittances (1 = yes)	0.045***	− 0.006	0.002	0.011
	(0.017)	(0.006)	(0.006)	(0.008)
Observations	24,805	45,121	37,822	18,932
Adjusted *R*^2^	0.097	0.094	0.115	0.118

Source: Authors’ estimation based on Gallup World Poll data

Notes: Robust standard errors in parentheses. ***p < 0.01, **p < 0.05, *p < 0.1. Country- and year-fixed effects and individual controls are included in all regressions. Full econometric output is available upon request. See Table 12 of the Appendix for country group lists

Next, panel B of Table 2 reports the country income group results for positive affect. Both migration-related variables are positive and statistically significant in lower-middle-income countries. The relatives abroad variable is also positive and marginally significant (at the 10% level) in the upper-middle-income countries. In lower-middle-income and high-income countries, the emigration of household members is associated with above-average stress levels (panel C), albeit being only marginally statistically significant, while remittances have no statistically significant association. The magnitude of the coefficient estimate is somewhat higher in high-income countries, possibly because the pain of separation hits respondents harder in high- rather than in low-income countries. This could be explained by the relatively strong informal networks, extended family structures and norms related to raising children by non-biological parents in poorer countries (Mazzucato et al. 2015; Murphy, 2008), which may make it easier to deal with the negative emotions associated with being left behind.

In addition, remittance-receiving households in high-income countries report more depression experiences than their non-remittance receiving counterparts (panel D), possibly because receiving remittances in prosperous countries with relatively generous welfare systems is a marker of destitution or disadvantage and—as such—is accompanied by depression.^13^

Our results thus far suggest that the out-migration of family members enhances life evaluation through remittances in poor countries and through the residual migration effect in rich countries. To further examine the role income, we report results by within-country income group in Table 3. Panel A unequivocally supports the conclusion that remittances matter in poorer contexts, while the psychological well-being derived from knowing that family members have better opportunities abroad matter in rich contexts (within countries).

**Table 3:** Emigration of family members, remittances and psychological well-being of those staying behind, by within-country income quintiles, 2009–2011

	Quintile 1 (poorest)	Quintile 2	Quintile 3	Quintile 4	Quintile 5 (richest)
	Panel A: best possible life (0/10)				
Relatives abroad (1 = yes)	0.042	0.088**	0.046	0.071**	0.109***
	(0.047)	(0.041)	(0.038)	(0.035)	(0.031)
Remittances (1 = yes)	0.222***	0.096*	0.220***	0.118***	− 0.007
	(0.062)	(0.053)	(0.050)	(0.044)	(0.038)
Observations	24,271	25,436	25,242	26,382	28,552
Adjusted *R*^2^	0.259	0.254	0.264	0.273	0.254
	Panel B: positive affect index (0/10)				
Relatives abroad (1 = yes)	0.174**	0.141*	0.028	0.175***	0.027
	(0.080)	(0.076)	(0.072)	(0.065)	(0.056)
Remittances (1 = yes)	− 0.029	0.156	0.209**	0.031	0.047
	(0.111)	(0.099)	(0.090)	(0.080)	(0.068)
Observations	20,655	21,413	21,597	22,386	24,269
Adjusted *R*^2^	0.244	0.215	0.191	0.183	0.148
	Panel C: stress yesterday (0/1)				
Relatives abroad (1 = yes)	0.016	0.004	0.013	0.018**	0.004
	(0.010)	(0.009)	(0.009)	(0.009)	(0.008)
Remittances (1 = yes)	0.008	− 0.005	− 0.016	0.003	−0.004
	(0.014)	(0.012)	(0.012)	(0.010)	(0.009)
Observations	21,559	22,296	22,468	23,277	25,284
Adjusted *R*^2^	0.131	0.123	0.106	0.092	0.105
	Panel D: depressed yesterday (0/1)				
Relatives abroad (1 = yes)	0.012	0.006	0.014*	0.011*	0.005
	(0.009)	(0.008)	(0.007)	(0.006)	(0.006)
Remittances (1 = yes)	− 0.015	− 0.004	− 0.005	0.011	0.005
	(0.011)	(0.010)	(0.009)	(0.008)	(0.007)
Observations	21,539	22,272	22,435	23,269	25,262
Adjusted *R*^2^	0.135	0.106	0.097	0.078	0.071

Source: Authors’ estimation based on Gallup World Poll data

Robust standard errors in parentheses. ***p < 0.01, **p < 0.05, *p < 0.1. Country- and year-fixed effects and individual controls are included in all regressions. Full econometric output is available on request. See Tables 11 and 12 in the Appendix for variable definitions and the list of countries included in each survey wave

Table 3 provides some further nuances in our findings. For example, remittances do not seem to matter for positive emotions across the income quintiles, but the residual migration effect matters in all quintiles except for the richest people within a country (panel B). Remittances are unassociated with stress and depression, but the pain of separation is concentrated among respondents in the middle-income quintiles.^14^^,^^15^

### Further heterogeneity analyses

Given the income findings reported above, we also investigated whether the relationship between emigration of household members and the subjective well-being of the left behind depends on how unequal a society is. The results by income inequality group, reported in Table 4, demonstrate that remittances are associated with evaluative well-being (measured as evaluations of the best possible life (BPL)) in more unequal countries, which could reflect the capabilities-enhancing role of remittances where social redistribution systems are weak and supports the income findings reported above. Furthermore, the analysis suggests that the emigration of family members is associated with higher levels of depression in more unequal countries. It is possible that in such contexts, where social cohesion and public support systems are weaker than in more equal societies, migrants find it particularly difficult to cope with the pain of separation.

**Table 4:** Emigration of family members, remittances, and psychological well-being of those staying behind, by income inequality (Gini coefficient) quartiles, 2009–2011

	Quartile 1 (most equal countries)	Quartile 2	Quartile 3	Quartile 4 (most unequal countries)
	Panel A: best possible life (0/10)			
Relatives abroad (1 = yes)	0.053*	0.072**	0.042	0.123***
	(0.031)	(0.033)	(0.038)	(0.030)
Remittances (1 = yes)	0.037	0.042	0.190***	0.164***
	(0.037)	(0.043)	(0.044)	(0.040)
Observations	35,358	32,791	27,488	41,153
Adjusted *R*^2^	0.264	0.333	0.288	0.225
	Panel B: positive affect index (0/10)			
Relatives abroad (1 = yes)	0.186***	− 0.035	− 0.031	0.184***
	(0.064)	(0.071)	(0.072)	(0.043)
Remittances (1 = yes)	0.046	0.047	0.332***	− 0.012
	(0.079)	(0.096)	(0.080)	(0.057)
Observations	30,111	27,206	24,241	38,287
Adjusted *R*^2^	0.191	0.213	0.208	0.121
	Panel C: stress yesterday (0/1)			
Relatives abroad (1 = yes)	0.014*	0.002	0.021**	0.006
	(0.008)	(0.009)	(0.009)	(0.006)
Remittances (1 = yes)	− 0.004	− 0.007	− 0.020**	0.004
	(0.009)	(0.011)	(0.010)	(0.008)
Observations	32,269	28,076	25,451	39,115
Adjusted *R*^2^	0.091	0.100	0.139	0.112
	Panel D: depressed yesterday (0/1)			
Relatives abroad (1 = yes)	− 0.006	0.007	0.017**	0.014***
	(0.006)	(0.006)	(0.007)	(0.005)
Remittances (1 = yes)	0.006	− 0.009	0.008	− 0.002
	(0.007)	(0.009)	(0.008)	(0.007)
Observations	32,222	28,050	25,429	39,088
Adjusted *R*^2^	0.115	0.104	0.090	0.112

Source: Authors’ estimation based on Gallup World Poll data

Robust standard errors in parentheses. ***p < 0.01, **p < 0.05, *p < 0.1. Country- and year-fixed effects and individual controls are included in all regressions. Country classifications are based on Gini coefficient data from the WDI and UNU-WIDER World Income Inequality Database, 2007–2011. The quartiles are as follows: *1* = first quartile (most equal countries, GINI between 24 and 32.13); *2* second quartile (GINI between 32.84 and 37); *3* third quartile (GINI between 37.19 and 45); *4* fourth quartile (most unequal countries, GINI between 45.13 and 63). Full econometric output is available on request. See Table 12 of the Appendix for the list of countries in each category

Next, Table 5 presents the results according to the country-level net migration rate, based on the United Nations data for 2005–2010. Panel A documents that having relatives abroad is positively associated with life evaluations in countries with lower emigration rate quartiles. Remittances are positively associated with BPL in countries with high emigration rates. This finding reflects our earlier result that remittances are particularly important for evaluative well-being in lower-income countries, where out-migration rates tend to be high. In countries with relatively low emigration rates, remittances are even negatively associated with BPL (quartile 3)^16^ or not associated with BPL (quartile 4). We also find that migrant relatives are more likely to experience stress and depression in countries with relatively low emigration rates (quartiles 2–4), while the coefficients are insignificant in high-emigration countries (quartile 1). A possible explanation is that people in high-emigration countries have developed mechanisms to deal with the negative consequences of emigration. By contrast, where emigration is less common, people have less knowledge of how to cope when someone leaves. In addition, the lower subjective well-being benefits of emigration in countries with lower emigration rates could reflect stigma attached to emigration—it is not the social norm to leave or receive migrant remittances.

**Table 5:** Emigration of family members, remittances, and psychological well-being of those staying behind, by net migration rate quartile, 2009–2011

	Quartile 1 (highest net migration rate)	Quartile 2	Quartile 3	Quartile 4 (lowest net migration rate)
	Panel A: best possible life (0/10)			
Relatives abroad (1 = yes)	0.049	0.082***	0.115***	0.105***
	(0.032)	(0.027)	(0.033)	(0.040)
Remittances (1 = yes)	0.213***	0.131***	−0.109**	0.080
	(0.038)	(0.032)	(0.044)	(0.076)
Observations	28,594	45,344	42,776	25,754
Adjusted *R*^2^	0.219	0.228	0.299	0.304
	Panel B: positive affect index (0/10)			
Relatives abroad (1 = yes)	0.141**	0.161***	0.020	0.015
	(0.058)	(0.048)	(0.056)	(0.094)
Remittances (1 = yes)	0.142**	− 0.038	0.151**	0.088
	(0.065)	(0.059)	(0.075)	(0.201)
Observations	23,004	40,419	39,983	18,201
Adjusted *R*^2^	0.213	0.210	0.190	0.162
	Panel C: stress yesterday (0/1)			
Relatives abroad (1 = yes)	0.004	0.014**	0.003	0.025*
	(0.008)	(0.006)	(0.007)	(0.013)
Remittances (1 = yes)	− 0.013	0.005	− 0.008	0.013
	(0.009)	(0.007)	(0.009)	(0.027)
Observations	23,810	42,268	41,695	19,030
Adjusted *R*^2^	0.142	0.095	0.100	0.108
	Panel D: depressed yesterday (0/1)			
Relatives abroad (1 = yes)	− 0.002	0.013**	0.018***	− 0.001
	(0.006)	(0.005)	(0.006)	(0.008)
Remittances (1 = yes)	0.006	0.000	− 0.006	0.031
	(0.007)	(0.006)	(0.008)	(0.020)
Observations	23,795	42,187	41,674	19,024
Adjusted *R*^2^	0.107	0.110	0.102	0.082

Source: Authors’ estimation based on Gallup World Poll data

Notes: Robust standard errors in parentheses. ***p < 0.01, **p < 0.05, *p < 0.1. Country- and year-fixed effects, and individual controls are included in all regressions. Tables 11 and 12 include variable definitions and the list of countries included in each survey wave

### Robustness checks

We performed several robustness checks. First, we wanted to understand to what extent the main findings are influenced by the sample composition of countries across the years and whether the availability of some countries in more than 1 year biases the findings. Specifically, since we limit the sample to when both the relatives abroad and the *remittances* variables are non-missing, our main estimation sample spans the years 2009–2011. In addition, while our 2009 sample comprises 112 countries, only 26 countries (located in Latin America and the Western Balkans) and 7 countries (located in the Western Balkans) could be included in the 2010 and 2011 analyses, respectively (see Table 12 in the Appendix). While we are limited by data availability, we offer a series of robustness checks that demonstrate that sample composition is not the driver of our main findings and conclusions.

First, we furnish specifications using data for 2009 only, which are not substantively different from the full sample (2009–2011) results (Table 6). Second, we have also separately estimated the models for the seven Western Balkans countries, the only country group that appears in all 3 years. The results shown in Table 7 demonstrate that the coefficient estimates on the key variables are mostly statistically insignificant or sufficiently different from those in the full sample (Table 1), meaning that the inclusion of the Western Balkan countries in 3 years does not drive the main estimates. This is true regardless of whether we estimate these regressions with country and year dummies or with country × year fixed effects (Table 7 panel A vs. panel B). Finally, we also conducted weighted regressions, whereby observations from countries that appear in the regressions just once are given a weight of 1, observations from countries that appear in the regressions twice receive a weight of 0.5, and observations from countries that appear in the regressions three times, receive a weight of 0.33. The results, presented in Table 8, do not differ substantively from the main findings reported in Table 1. In summary, the series of checks presented in Tables 6–8 provide evidence that our results are not biased because of the greater availability of some countries compared to others.

**Table 6:** Emigration of family members, remittances, and psychological well-being of those staying behind, ordinary least squares results, 2009 only

	BPL (0/10)	Positive affect (0/10)	Stress (0/1)	Depressed (0/1)
	(1)	(2)	(3)	(4)
Relatives abroad	0.072***	0.068*	0.010**	0.007*
	(0.018)	(0.036)	(0.005)	(0.004)
Remittances	0.102***	0.136***	− 0.001	0.007
	(0.024)	(0.045)	(0.006)	(0.005)
Country and survey wave Dummies	Yes	Yes	Yes	Yes
Observations	111,561	91,958	96,052	95,946
Adjusted *R*^2^	0.297	0.199	0.119	0.108

Source: Authors’ estimation based on Gallup World Poll data

Notes: Robust standard errors in parentheses. ***p < 0.01, **p < 0.05, *p < 0.1. Country- and year-fixed effects and individual controls are included in all regressions. Full econometric output is available upon request

**Table 7:** Emigration of family members, remittances, and psychological well-being of those staying behind, Western Balkans, ordinary least squares results, 2009–2011

	BPL (0/10)	Positive affect (0/10)	Stress (0/1)	Depressed (0/1)
	Panel A			
	(1)	(3)	(5)	(7)
Relatives abroad	0.098**	0.064	0.026***	0.008
	(0.040)	(0.078)	(0.010)	(0.007)
Remittances	− 0.022	0.060	− 0.007	− 0.008
	(0.042)	(0.085)	(0.010)	(0.007)
Country and survey wave dummies	Yes	Yes	Yes	Yes
Observations	19,520	18,313	19,433	19,398
Adjusted *R*^2^	0.173	0.170	0.063	0.094
	Panel B			
Relatives abroad	0.114***	0.069	0.025***	0.008
	(0.040)	(0.077)	(0.010)	(0.007)
Remittances	−0.019	0.071	−0.008	−0.008
	(0.042)	(0.085)	(0.010)	(0.007)
Country and survey wave dummies	Yes	Yes	Yes	Yes
Country × survey wave dummies	Yes	Yes	Yes	Yes
Observations	19,520	18,313	19,433	19,398
Adjusted *R*^2^	0.183	0.174	0.065	0.096

Source: Authors’ estimation based on Gallup World Poll data

Notes: Robust standard errors in parentheses. ***p < 0.01, **p < 0.05, *p < 0.1. The Western Balkan countries are Albania, Bosnia and Herzegovina, Croatia, Macedonia, Montenegro, Serbia, and Kosovo. Full econometric output is available upon request

**Table 8:** Emigration of family members, remittances, and psychological well-being of those staying behind, full sample, ordinary least squares results, 2009–2011, weighted

	BPL (0/10)	Positive affect (0/10)	Stress (0/1)	Depressed (0/1)
	(1)	(2)	(3)	(4)
Relatives abroad	0.077***	0.095***	0.009**	0.007**
	(0.016)	(0.032)	(0.004)	(0.003)
Remittances	0.116***	0.112***	− 0.005	0.005
	(0.021)	(0.040)	(0.005)	(0.004)
Observations	142,468	121,607	126,803	126,680
Adjusted *R*^2^	0.298	0.198	0.117	0.104

Source: Authors’ estimation based on Gallup World Poll data

Notes: Robust standard errors in parentheses. ***p < 0.01, **p < 0.05, *p < 0.1. *Notes*: Robust standard errors in parentheses. ***p < 0.01, **p < 0.05, *p < 0.1. Country- and year-fixed effects, and individual controls are included in all regressions. Full econometric output is available upon request

A second concern related to our analysis is that the results we should could be driven by the selection of individuals into migration. First, there is selection into migration across households within the same country, and second, there is selection within the household members regarding which family member emigrates (Gibson et al. 2011). Using information on family members who were selected to emigrate from Tonga to New Zealand using a migration lottery, Gibson et al. (2011) compare experimental and non-experimental findings to assess to what extent selection is a problem. They conclude that while selection is an issue when comparing outcomes at the household level, selection is not a problem when examining individual-level outcomes, which is the case in our paper.

We are limited in our ability to tackle endogeneity issues directly, as explained above. Nevertheless, we provide some suggestive evidence on whether selection issues could be entirely driving our results. Specifically, we rely on a method that involves (i) a pre-processing step to create comparable groups of respondents with and without family members abroad using entropy balancing (Hainmueller 2012)^17^; and (ii) estimating a weighted regression using the entropy balancing weights generated in the pre-processing step whereby we regress having a family member abroad on subjective well-being and the other controls. Entropy balancing is similar to traditional statistical matching techniques but is arguably superior to them (Hainmueller 2012) while allowing us to also to mitigate issues related to selection into migration across households.^18^ The regressions using the entropy balancing weights are presented in Table 9. These findings deviate very little from the main findings presented in Table 1, suggesting that selection is not the primary driver behind our baseline results. Admittedly, we cannot say much on selection on unobservables using the entropy balancing method, but the findings in Table 9 provide some reassurance that selection is not the main mechanism behind the patterns we describe.^19^

**Table 9:** Emigration of family members, remittances and psychological well-being of those staying behind, full sample, ordinary least squares applied after entropy balancing, 2009–2011

	BPL (0/10)	Positive affect (0/10)	Stress (0/1)	Depressed (0/1)
	(1)	(2)	(3)	(4)
Relatives abroad	0.085***	0.099***	0.010**	0.011***
	(0.017)	(0.030)	(0.004)	(0.003)
Remittances	0.080***	0.083*	− 0.011*	− 0.006
	(0.025)	(0.045)	(0.006)	(0.005)
Observations	142,468	121,607	126,803	126,680
Adjusted *R*^2^	0.262	0.195	0.112	0.103

Source: Authors’ estimation based on Gallup World Poll data

Bootstrapped standard errors in parentheses. ***p < 0.01, **p < 0.05, *p < 0.1. ***p < 0.01, **p < 0.05, *p < 0.1. Country- and year-fixed effects, and individual controls are included in all regressions. Full econometric output is available on request

Our final robustness check, reported in Table 10, involves using a different independent variable, namely, having relatives and friends abroad (rather than having a relative abroad who left in the last 5 years as in the main specifications). Specifically, the variable is based on a question asking respondents: “Do you have relatives or friends who are living in another country whom you can count on to help you when you need them, or not?“(Gallup, Inc., 2005–2016). This variable is a closer measure of networks of friends and family members abroad rather than of left behind status (the correlation coefficient between the relatives abroad variable used in our main analyses, and this variable concerning networks abroad is 0.36). The results presented in Table 10 are similar to the ones in the main specification using the relatives abroad variable (Table 1).^20^

**Table 10:** Emigration of family members, remittances, and psychological well-being of those staying behind, full sample, ordinary least squares results, different key independent variable, 2009–2011

	BPL (0/10)	Positive affect (0/10)	Stress (0/1)	Depressed (0/1)
	(1)	(2)	(3)	(4)
Network of relatives and friends abroad	0.173***	0.278***	0.004	− 0.004*
	(0.013)	(0.023)	(0.003)	(0.002)
Remittances	0.041**	− 0.033	− 0.002	0.008**
	(0.021)	(0.037)	(0.005)	(0.004)
Observations	142,468	121,607	126,803	126,680
Adjusted *R*^2^	0.262	0.195	0.112	0.103

Source: Authors’ estimation based on Gallup World Poll data

Notes: Robust standard errors in parentheses. ***p < 0.01, **p < 0.05, *p < 0.1. Country- and year-fixed effects, and individual controls are included in all regressions. Full econometric output is available upon request

### Limitations

While we view this work as a step towards understanding the linkages between emigration and the different subjective well-being dimensions (evaluative well-being, positive hedonic well-being, stress, and depression) of those left behind in a wide range of origin countries, our results cannot be interpreted as causal. Panel data tracing migrants and those left behind over time or a credible instrument for the out-migration of family members could help to address some sources of endogeneity (specifically, time-invariant respondent heterogeneity) and represent directions for future research. However, this line of inquiry would involve a trade-off between the panel dimension and geographical breadth, as multi-country longitudinal surveys require large financial resources and hence are rare.

Another limitation that we acknowledge is that we lack information on (i) the relationship between the emigrated household member and the survey respondent (i.e., we do not know whether the emigrants are partners, children, parents, or siblings); (ii) what characteristics migrants have (age, gender, education, etc.); (iii) when exactly in the past 5 years the migrant left and whether they are abroad permanently or temporarily; and (iv) the exact type of migrant remittances (monetary or in-kind) and the monetary amount of the remittances. The fact that we have relatively “recent” migrants—rather than those who have been living abroad for more than 5 years—suggests that we are capturing the short- to medium-run well-being consequences of emigration for the relatives left behind. Yet, the associations we document are small in magnitude. We expect that our results would be less significant or even smaller in magnitude if households with migrants who left a long time ago were considered, given that these households would have had sufficient time to adjust to and cope with being left behind.

While the Gallup World Poll is remarkably detailed, it has not been specifically designed to study migration and is not a household-level survey. If possible, future research should consider migrant characteristics, given that they may have a differential impact on the well-being of those staying behind. Practically, the level of detail available in a survey would again need to be weighed against its geographical breadth.

Finally, given that our analysis samples cover only the adult population in each country, we do not have information on young children under age 15 who are left behind. Nonetheless, the literature highlights that the issue of left-behind children should be devoted particular attention especially given the increasing number of female labor migrants.

## Conclusion

This paper examined the broad subjective well-being consequences of migration for migrant relatives staying behind, as reflected in life evaluations, positive and negative emotions, and depression experiences. Using 2009–2011 Gallup World Poll data for 114 countries, we are the first to explore the association between emigration and the subjective well-being of those staying behind across a wide range of sending countries. We find that people with family members abroad have higher levels of evaluative well-being (BPL) and positive affect than people from non-migrant households. At the same time, those staying behind are also more likely to experience stress and depression. Migrant remittances appear to amplify the positive associations related to evaluative well-being and positive emotions but do not contribute to reducing stress and depression. Overall, these findings suggest that the emigration of family members is likely to trigger a range of subjective well-being responses among those staying behind, with remittances reinforcing the positive outcomes (life evaluations and positive affect) but not offsetting the negative ones (stress and depression). In other words, remittances buy “happiness” but do not relieve the pain of separation.

We also find that the level of economic prosperity and equality—both across and within countries—is important for understanding the relationship between migration-related variables and the well-being of those staying behind. One of the most distinct results of this study is that remittances are particularly beneficial for life evaluations in poorer and more unequal countries, as well as among poorer respondents within a particular country, probably because remittances increase the opportunities and capabilities of respondents in such circumstances. Among richer countries, meanwhile, only the emigration of family members is positively associated with life evaluations, while remittances have no additional association. Higher depression and stress levels are also reported among migrant relatives and remittance recipients in more developed countries, which is a previously undocumented result.

Finally, we find that the emigration of family members is associated with lower stress and depression in countries with higher emigration rates. It is possible that people in these countries have developed mechanisms to cope with the negative psychological effects of emigration such as the pain of separation. In countries where emigration is less common, decision-makers design policies to mitigate the negative experiences of stress and depression for vulnerable groups of household members staying behind. Some societies and communities in which migration is common already have informal groups in place that help with information sharing or preparing for migration (Cattaneo 2015; Gallego and Mendola, 2013). Facilitating such formal or informal support groups and stress and depression prevention programs for those with household members abroad could be socially beneficial.

Overall, our findings demonstrate the role of emigration and remittances for subjective well-being of those staying behind in poorer countries. However, while the out-migration of family members and remittances could improve well-being through easing budget constraints and providing financial security, it is a complement rather than a substitute for economic and institutional development.

## Appendix

**Table 11:** Variable definitions

Variable	Explanation
Dependent variables	
Evaluative well-being: best possible life (BPL) (0/10)	The respondent’s assessment (on a 0–10 scale) based on the Cantril ladder of life question, whereby respondents are asked to imagine a ladder numbered from 0 at the bottom and 10 at the top. 0 represents the worst possible life they can imagine for themselves, while 10 represents the best possible life.
Positive affect index (0/10)	An index of positive affect/emotions based on yes/no questions about whether the respondent experienced a lot of happiness yesterday, smiled a lot yesterday, and whether she experienced joy yesterday. Constructed using principal component analysis and re-scaled to range from 0 (no positive affect) to 10 (a lot of positive affect).
Stress yesterday (0/1)	A binary indicator coded as 1 if the respondent experienced a lot of stress the previous day and 0 otherwise.
Depressed yesterday (0/1)	A binary indicator variable coded as 1 if the respondent experienced a lot of depression the previous day and 0 otherwise.
Focal independent variables	
Relative abroad (1 = yes)	A binary indicator variable coded as 1 if the respondent answered that any members of the household have gone to live in a foreign country permanently or temporarily in the past 5 years and are still living there. Respondents who have family members who are still there are coded as 1 and those with family members who returned from abroad and no family members abroad in the past 5 years are coded as 0.
Remittances (1 = yes)	A binary indicator variable based on the question of whether the respondent’s household received help in the form of money or goods from another individual in the past 12 months. The variable takes the value of 1 for respondents receive money or goods from an individual abroad and both abroad and from this country, and zero otherwise.
Other controls	
Within-country household income quintile indicators	This variable is based on the Gallup-provided within-country household income quintile variable, whereby 1 corresponds to the poorest 20%; 5 corresponds to the richest 20%, and 6 is an indicator for missing information.
Education level	Elementary education: completed elementary education or less (up to 8 years of basic education); Secondary education: completed secondary education or up to 3 years of tertiary education (9 to 15 years of education); Tertiary education: completed 4 years of education beyond “high school” and/or received a 4-year college degree
Pain yesterday	A binary indicator variable coded as 1 if the respondent experienced a lot of physical pain the day before and 0 if they did not.
Satisfaction with personal health	A binary indicator variable, which is coded as 1 if the respondent indicated that he or she is satisfied with their personal health and 0 if they responded that they are dissatisfied.
Health problem	A binary indicator coded as 1 if respondents have health problems that prevent them from doing any of the things people their age normally can do and 0 if they do not have such problems.
Household and demographic variables	Age, gender, household size, indicator for presence of children in the household, religiosity, marital status, urban/rural location dummy, and employment status. Note that religiosity is a binary indicator for whether religion is important in the respondent’s life.

Source: Gallup World Poll Documentation (Gallup Inc., 2005-2018)

Notes: To prevent non-random attrition bias due to missing data, we included indicator dummies for missing information for all variables. The questions pertain to Gallup: Copyright © 2005–2018 Gallup, Inc.

**Table 12:** Countries included in the analyses, by year and country group

	2009		2010	2011
Countries according to year of interview	Afghanistan, Albania, Argentina, Armenia, Azerbaijan, Bahrain, Bangladesh, Belarus, Bolivia, Bosnia and Herzegovina, Brazil, Burundi, Cambodia, Cameroon, Canada, Chad, Chile, China, Colombia, Comoros, Congo (Kinshasa), Costa Rica, Croatia, Cyprus, Denmark, Djibouti, Dominican Republic, Ecuador, Egypt, Estonia, France, Georgia, Germany, Ghana, Greece, Guatemala, Honduras, Hong Kong, India, Indonesia, Iraq, Ireland, Israel, Italy, Ivory Coast, Japan, Jordan, Kazakhstan, Kenya, Kosovo, Kuwait, Kyrgyzstan, Latvia, Lebanon, Lithuania, Macedonia, Malawi, Malaysia, Mali, Mauritania, Mexico, Moldova, Montenegro, Nepal, Nicaragua, Niger, Nigeria, Pakistan, Palestinian Territories, Panama, Paraguay, Peru, Philippines, Romania, Russia, Rwanda, Saudi Arabia, Senegal, Serbia, Singapore, Slovenia, South Africa, South Korea, Spain, Sri Lanka, Sudan, Sweden, Switzerland, Syria, Tajikistan, Tanzania, Thailand, Tunisia, Turkey, Turkmenistan, Uganda, Ukraine, United Arab Emirates, United Kingdom, United States of America, Uruguay, Uzbekistan, Venezuela, Vietnam, Yemen, Zambia, and Zimbabwe		Albania, Argentina, Austria, Bolivia, Bosnia and Herzegovina, Brazil, Bulgaria, Chile, Colombia, Costa Rica, Croatia, Czech Republic, Dominican Republic, Ecuador, El Salvador, Guatemala, Haiti, Honduras, Kosovo ,Macedonia, Mexico, Montenegro, Nicaragua, Panama, Paraguay, Peru, Poland, Portugal, Serbia, Uruguay, and Venezuela	Albania, Bosnia and Herzegovina, Croatia, Kosovo, Macedonia, Montenegro, and Serbia
Countries according to World Bank income group, 2010	High income	Upper-middle income	Lower-middle income	Low income
	Austria, Bahrain, Canada, Croatia, Cyprus, Czech Republic, Denmark, Estonia, France, Germany, Greece, Hong Kong, Ireland, Israel, Italy, Japan, Kuwait, Latvia, Poland, Portugal, Saudi Arabia, Singapore, Slovenia, South Korea, Spain, Sweden, Switzerland, United Arab Emirates, United Kingdom, and United States of America	Albania, Argentina, Azerbaijan, Belarus, Bosnia and Herzegovina, Brazil, Bulgaria, Chile, Colombia, Costa Rica, Dominican Republic, Kazakhstan, Lebanon, Lithuania, Macedonia, Malaysia, Mexico, Montenegro, Panama, Peru, Romania, Russia, Serbia, South Africa, Turkey, Uruguay, and Venezuela	Armenia, Bolivia, Cameroon, China, Djibouti, Ecuador, Egypt, El Salvador, Georgia, Guatemala, Honduras, India, Indonesia, Iraq, Ivory Coast, Jordan, Kosovo, Moldova, Nicaragua, Nigeria, Pakistan, Palestinian Territories, Paraguay, Philippines, Senegal, Sri Lanka, Sudan, Syria, Thailand, Tunisia, Turkmenistan, Ukraine, Uzbekistan, Vietnam, and Yemen	Afghanistan, Bangladesh, Burundi, Cambodia, Chad, Comoros, Congo (Kinshasa), Ghana, Haiti, Kenya, Kyrgyzstan, Malawi, Mali, Mauritania, Nepal, Niger, Rwanda, Tajikistan, Tanzania, Uganda, Zambia, and Zimbabwe
Countries according to income inequality quartile (based on 2007–2011 Gini coefficient data); (source: World Bank World Development Indicators and UNU-WIDER World Income Inequality Database	Quartile 1 (most equal countries)	Quartile 2	Quartile 3	Quartile 4 (most unequal countries)
	Egypt, Syria, Pakistan, Bangladesh, Germany, Czech Republic, Sweden, Denmark, Japan, Afghanistan, Belarus, Kazakhstan, Kyrgyzstan, Ukraine, Albania, Armenia, Austria, Azerbaijan, Croatia, Cyprus, Estonia, Iraq, Ireland, Montenegro, Serbia, Slovenia, Tajikistan, and Kosovo	Lebanon, Indonesia, United Kingdom, France, Spain, Italy, Poland, Romania, Greece, India, Venezuela, Palestinian Territories, Canada, Sri Lanka, Cambodia, Mali, Mauritania, Niger, South Korea, Moldova, Bosnia, and Herzegovina, Bulgaria, Burundi, Latvia, Lithuania, Nepal, Portugal, Sudan, Switzerland, and Tunisia	United States of America, Jordan, Turkey, China, Nigeria, Tanzania, Israel, Uganda, Philippines, Vietnam, Thailand, Senegal, Georgia, Russia, Cameroon, Zimbabwe, Chad, Congo (Kinshasa), El Salvador, Ivory Coast, Macedonia, Uzbekistan, and Yemen	Hong Kong, Singapore, Brazil, Mexico, Kenya, Malawi, South Africa, Rwanda, Zambia, Costa Rica, Argentina, Bolivia, Chile, Colombia, Comoros, Djibouti, Dominican Republic, Ecuador, Guatemala, Haiti, Honduras, Malaysia, Nicaragua, Panama, Paraguay, Peru, and Uruguay
Countries according to net migration rates, 2005–2010 (source: United Nations Population Division)	Quartile 1 (highest net migration rate)	Quartile 2	Quartile 3	Quartile 4 (lowest net migration rate)
	Afghanistan, Albania, Armenia, Bangladesh, Cambodia, Comoros, Djibouti, Dominican Republic, El Salvador, Georgia, Iraq, Latvia, Lithuania, Nepal, Nicaragua, Palestinian Territories, Paraguay, Peru, Philippines, Romania, Sri Lanka, Sudan, Uruguay, and Zimbabwe	Azerbaijan, Bolivia Bulgaria, Cameroon, Colombia, Croatia, Egypt, Estonia, Guatemala, Haiti, Honduras, Indonesia, Ivory Coast, Kenya, Kosovo, Kyrgyzstan, Mali, Mauritania, Mexico, Moldova, Montenegro, Pakistan, Rwanda, Senegal, Serbia, Tajikistan Tanzania, Thailand, Tunisia, Turkmenistan, Uganda, Uzbekistan, Vietnam, and Zambia	Argentina, Belarus, Bosnia and Herzegovina, Brazil, Chad, Chile, China, Congo (Kinshasa), Costa Rica, Ecuador, France, Germany, Ghana, Greece, Hong Kong, India, Japan, Kazakhstan, Macedonia, Malawi, Niger, Nigeria, Panama, Poland, South Korea, Turkey, Ukraine, Venezuela, and Yemen	Austria, Bahrain, Burundi, Canada, Cyprus, Czech Republic, Denmark, Ireland, Israel, Italy, Jordan, Kuwait, Lebanon, Malaysia, Portugal, Russia, Saudi Arabia, Singapore, Slovenia, South Africa, Spain, Sweden, Switzerland, Syria, United Arab Emirates, United Kingdom, United States of America

**Table 13:** Summary statistics of analysis variables

Variable	Overall		
	*N*	Mean	Std. Dev.
Best possible life (0 = worst, 10 = best)	142,468	5.495	2.213
Positive affect index (0/10)	121,607	7.205	3.571
Stressed yesterday (0/1)	126,803	0.259	0.438
Depressed yesterday (0/1)	126,680	0.124	0.329
Received remittances	144,003	0.084	0.278
Age: 15–35 years	122,690	5.363	3.505
Age 36–60 years	144,003	0.458	0.498
Age over 60 years	144,003	0.390	0.488
Age missing	144,003	0.148	0.355
Female	144,003	0.539	0.498
Marital status: married or living with partner	144,003	0.581	0.493
Unmarried	144,003	0.415	0.493
Marital status missing	144,003	0.004	0.064
Children in the household: none	144,003	0.419	0.493
Children in household	144,003	0.515	0.500
Children information missing	144,003	0.066	0.249
Household size	144,003	4.537	2.844
Per capita household. Income: first quintile	144,003	0.171	0.376
Second quintile	144,003	0.178	0.383
Third quintile	144,003	0.177	0.381
Fourth quintile	144,003	0.185	0.388
Fifth quintile	144,003	0.201	0.401
Household income missing	144,003	0.089	0.284
Education: elementary education	144,003	0.332	0.471
Secondary	144,003	0.528	0.499
Tertiary	144,003	0.129	0.336
Education missing	144,003	0.010	0.102
Employment Status: employed	144,003	0.482	0.500
Unemployed	144,003	0.043	0.203
Out of the labor force	144,003	0.362	0.481
Employment status missing	144,003	0.113	0.317
Pain yesterday: none	144,003	0.724	0.447
Pain yesterday	144,003	0.266	0.442
Pain yesterday missing	144,003	0.010	0.098
Personal health: satisfied	144,003	0.773	0.419
Dissatisfied	144,003	0.217	0.412
Personal health missing	144,003	0.010	0.099
Health problem: none	144,003	0.740	0.438
Has a health problem	144,003	0.251	0.434
Health problem missing	144,003	0.008	0.091
Religiosity: religion important	144,003	0.718	0.450
Religion not important	144,003	0.233	0.423
Religiosity missing	144,003	0.049	0.215
Household location: small city/village	144,003	0.531	0.499
Large city	144,003	0.423	0.494
Location missing	144,003	0.045	0.208

Source: Authors’ estimation based on Gallup World Poll data

Note: See Table 11 in the Appendix for variable definitions and Table 12 for the included countries

**Table 14:** Emigration of family members, remittances, and psychological well-being of those staying behind, by education group, ordinary least squares results, 2009–2011

	Elementary	Secondary	Tertiary
Panel A: best possible life (0/10)			
Relatives abroad (1 = yes)	0.121***	0.051**	0.096***
	(0.033)	(0.021)	(0.037)
Remittances (1 = yes)	0.244***	0.045*	0.032
	(0.040)	(0.027)	(0.053)
Observations	47,263	75,293	18,427
Adjusted *R*^2^	0.223	0.271	0.262
Panel B: positive affect index (0/10)			
Relatives abroad (1 = yes)	0.175***	0.038	0.163**
	(0.056)	(0.040)	(0.071)
Remittances (1 = yes)	0.043	0.158***	− 0.105
	(0.069)	(0.050)	(0.098)
Observations	40,577	64,007	15,593
Adjusted *R*^2^	0.231	0.188	0.149
Panel C: stress yesterday (0/1)			
Relatives abroad (1 = yes)	0.014**	0.006	0.011
	(0.007)	(0.005)	(0.010)
Remittances (1 = yes)	− 0.027***	0.009	0.002
	(0.008)	(0.006)	(0.013)
Observations	41,878	67,012	16,442
Adjusted *R*^2^	0.120	0.106	0.100
Panel D: depressed yesterday (0/1)			
Relatives abroad (1 = yes)	0.009	0.012***	− 0.003
	(0.006)	(0.004)	(0.007)
Remittances (1 = yes)	0.003	0.001	0.010
	(0.007)	(0.005)	(0.009)
Observations	41,854	66,937	16,424
Adjusted *R*^2^	0.125	0.085	0.081

Source: Authors’ estimation based on Gallup World Poll data

Notes: Robust standard errors in parentheses. ****p* < 0.01, ***p* < 0.05, **p* < 0.1. Country- and year-fixed effects and individual controls are included in all regressions. Full econometric output available upon request

**Table 15:** Descriptive statistics, selected variables, before and after entropy balancing

	Relatives abroad		No relatives abroad unmatched		No relatives abroad matched		Standardized bias %	
	Mean	Variance	Mean	Variance	Mean	Variance	Unmatched	Matched
Ages 36–60	0.354	0.229	0.396	0.239	0.354	0.229	− 0.089	0.000
Over 60	0.143	0.123	0.148	0.126	0.143	0.123	− 0.015	0.000
Age missing	0.003	0.003	0.004	0.004	0.003	0.003	− 0.035	0.000
Female	0.534	0.249	0.540	0.248	0.534	0.249	− 0.011	0.000
Married/living with partner	0.548	0.248	0.586	0.243	0.548	0.248	− 0.078	0.000
Marital status missing	0.003	0.003	0.004	0.004	0.003	0.003	− 0.028	0.000
Children in household	0.544	0.248	0.510	0.250	0.544	0.248	0.068	0.000
Children information missing	0.075	0.069	0.065	0.061	0.075	0.069	0.039	0.000
Household size	4.834	9.363	4.487	7.857	4.833	9.362	0.113	0.000
Household size^2^	32.725	2896.166	27.994	2020.589	32.724	2896.099	0.088	0.000
Second income quintile	0.224	0.174	0.194	0.157	0.224	0.174	0.072	0.000
Third income quintile	0.226	0.175	0.194	0.157	0.226	0.175	0.076	0.000
Fourth income quintile	0.192	0.155	0.200	0.160	0.192	0.155	− 0.019	0.000
Richest 20%	0.160	0.135	0.204	0.163	0.160	0.135	− 0.119	0.000
Income missing	0.018	0.018	0.005	0.005	0.018	0.018	0.097	0.000
Secondary education	0.540	0.248	0.526	0.249	0.540	0.248	0.028	0.000
Tertiary education	0.153	0.130	0.126	0.110	0.153	0.130	0.076	0.000
Education missing	0.012	0.012	0.010	0.010	0.012	0.012	0.018	0.000
Unemployed	0.050	0.048	0.042	0.040	0.050	0.048	0.039	0.000
Out of the labor force	0.367	0.232	0.361	0.231	0.367	0.232	0.012	0.000
Employment status missing	0.122	0.107	0.112	0.099	0.122	0.107	0.030	0.000
Pain yesterday	0.285	0.204	0.263	0.194	0.285	0.204	0.048	0.000
Pain information missing	0.009	0.009	0.010	0.010	0.009	0.009	− 0.002	0.000
Dissatisfied with health	0.215	0.169	0.217	0.170	0.215	0.169	− 0.004	0.000
Health satisfaction missing	0.011	0.011	0.010	0.010	0.011	0.011	0.009	0.000
Has a health problem	0.261	0.193	0.249	0.187	0.261	0.193	0.027	0.000
Health problem missing	0.007	0.007	0.009	0.009	0.007	0.007	− 0.019	0.000
Religion important	0.780	0.172	0.708	0.207	0.780	0.172	0.175	0.000
Religiosity missing	0.030	0.029	0.052	0.049	0.030	0.029	− 0.125	0.000
Large city	0.445	0.247	0.420	0.244	0.445	0.247	0.051	0.000
Location missing	0.058	0.055	0.043	0.041	0.058	0.055	0.063	0.000
Year 2010	0.220	0.172	0.162	0.135	0.220	0.172	0.142	0.000
Year 2011	0.047	0.045	0.046	0.044	0.047	0.045	0.003	0.000

Source: Authors’ estimation based on Gallup World Poll data

Notes: *N* = 144,003, *N* with family abroad = 20,649. The last two columns display the percent standardized bias, which is a measure of matching quality. It is calculated as the difference of the sample means in the treatment and the controls as a square root of the average of the sample variance in both groups

**Table 16:** Emigration, remittances and subjective well-being of those staying behind, full sample, ordinary least squares results, 2009–2011, without health controls

	BPL (0/10)	Positive affect (0/10)	Stress (0/1)	Depressed (0/1)
	(1)	(2)	(3)	(4)
Relatives abroad	0.070***	0.076**	0.013***	0.011***
	(0.016)	(0.030)	(0.004)	(0.003)
Remittances	0.105***	0.086**	−0.003	0.002
	(0.021)	(0.038)	(0.005)	(0.004)
Observations	142,468	121,607	126,803	126,680
Adjusted *R*^2^	0.256	0.133	0.060	0.042

Source: Authors’ estimation based on Gallup World Poll data

Notes: Robust standard errors in parentheses. ****p* < 0.01, ***p* < 0.05, **p* < 0.1. Country- and year-fixed effects and individual controls (except health controls) are included in all regressions. Full econometric output is available upon request

**Table 17:** Emigration of family members, remittances, and psychological well-being of those staying behind, non-missing observations only, ordinary least squares results, 2009–2011

	BPL (0/10)	Positive affect (0/10)	Stress (0/1)	Depressed (0/1)
	(1)	(2)	(3)	(4)
Relatives abroad	0.088***	0.079**	0.009**	0.007**
	(0.019)	(0.033)	(0.004)	(0.003)
Remittances	0.100***	0.083*	−0.005	0.002
	(0.024)	(0.042)	(0.005)	(0.004)
Observations	107,227	90,860	94,496	94,438
Adjusted *R*^2^	0.294	0.198	0.120	0.104

Source: Authors’ estimation based on Gallup World Poll data

Notes: Robust standard errors in parentheses. ****p* < 0.01, ***p* < 0.05, **p* < 0.1. Country- and year-fixed effects and individual controls (except health controls) are included in all regressions. Full econometric output is available upon request

**Table 18:** Correlates of having family member abroad and receiving remittances, logistic regressions, and average marginal effects

Variables	(1)	(2)	(3)	(4)	(5)	(6)	(7)	(8)
	Relatives abroad	Remittances	Relatives abroad	Remittances	Relatives abroad	Remittances	Relatives abroad	Remittances
Best possible life (0/10)	0.003***	0.003***						
	(0.000)	(0.000)						
Positive affect (0/10)			0.001***	0.001***				
			(0.000)	(0.000)				
Stress (0/1)					0.005**	0.000		
					(0.002)	(0.002)		
Depressed (0/1)							0.009***	0.003
							(0.003)	(0.002)
Ages 36–60	− 0.004**	− 0.003**	− 0.004	− 0.004**	− 0.005**	− 0.005***	− 0.005**	− 0.004**
	(0.002)	(0.002)	(0.002)	(0.002)	(0.002)	(0.002)	(0.002)	(0.002)
Over 60	0.021***	0.012***	0.021***	0.011***	0.021***	0.010***	0.021***	0.011***
	(0.004)	(0.003)	(0.004)	(0.003)	(0.004)	(0.003)	(0.004)	(0.003)
Female	0.002	0.007***	0.001	0.006***	0.001	0.006***	0.001	0.006***
	(0.002)	(0.001)	(0.002)	(0.002)	(0.002)	(0.002)	(0.002)	(0.002)
Married/living with partner	−0.011***	−0.016***	−0.013***	−0.015***	−0.012***	−0.015***	−0.012***	−0.015***
	(0.002)	(0.002)	(0.002)	(0.002)	(0.002)	(0.002)	(0.002)	(0.002)
Children in household	0.002	0.006***	0.005**	0.007***	0.005*	0.006***	0.004*	0.006***
	(0.002)	(0.002)	(0.003)	(0.002)	(0.003)	(0.002)	(0.003)	(0.002)
Household size	−0.003***	0.001*	−0.003***	0.001	−0.002***	0.001**	−0.002***	0.001**
	(0.001)	(0.001)	(0.001)	(0.001)	(0.001)	(0.001)	(0.001)	(0.001)
Household size^2^	0.021***	0.001	0.020***	0.002	0.020***	0.001	0.019***	0.001
	(0.004)	(0.003)	(0.004)	(0.003)	(0.004)	(0.003)	(0.004)	(0.003)
Second Income quintile	0.008***	0.009***	0.009***	0.010***	0.008***	0.010***	0.009***	0.010***
	(0.003)	(0.002)	(0.003)	(0.002)	(0.003)	(0.002)	(0.003)	(0.002)
Third income quintile	0.021***	0.020***	0.022***	0.022***	0.022***	0.022***	0.023***	0.021***
	(0.003)	(0.002)	(0.003)	(0.002)	(0.003)	(0.002)	(0.003)	(0.002)
Fourth income quintile	0.031***	0.035***	0.033***	0.037***	0.032***	0.037***	0.033***	0.036***
	(0.003)	(0.002)	(0.003)	(0.003)	(0.003)	(0.002)	(0.003)	(0.002)
Richest 20%	0.061***	0.059***	0.062***	0.062***	0.061***	0.061***	0.062***	0.060***
	(0.003)	(0.003)	(0.003)	(0.003)	(0.003)	(0.003)	(0.003)	(0.003)
Secondary education	0.023***	0.010***	0.021***	0.012***	0.021***	0.012***	0.022***	0.012***
	(0.002)	(0.002)	(0.002)	(0.002)	(0.002)	(0.002)	(0.002)	(0.002)
Tertiary education	0.040***	0.009***	0.036***	0.011***	0.037***	0.011***	0.037***	0.011***
	(0.003)	(0.003)	(0.004)	(0.003)	(0.004)	(0.003)	(0.004)	(0.003)
Unemployed	0.014***	0.006*	0.010**	0.006*	0.008*	0.006	0.008*	0.005
	(0.005)	(0.004)	(0.005)	(0.004)	(0.005)	(0.004)	(0.005)	(0.004)
Pain yesterday	0.007***	0.001	0.005**	0.000	0.003	−0.000	0.003	−0.001
	(0.002)	(0.002)	(0.002)	(0.002)	(0.002)	(0.002)	(0.002)	(0.002)
Dissatisfied with health	0.002	−0.001	0.001	−0.002	−0.002	−0.002	−0.002	−0.003
	(0.003)	(0.002)	(0.003)	(0.002)	(0.003)	(0.002)	(0.003)	(0.002)
Has a health problem	0.010***	0.009***	0.012***	0.009***	0.012***	0.009***	0.011***	0.009***
	(0.002)	(0.002)	(0.003)	(0.002)	(0.003)	(0.002)	(0.003)	(0.002)
Religion important	0.010***	0.006***	0.010***	0.009***	0.011***	0.008***	0.010***	0.008***
	(0.003)	(0.002)	(0.003)	(0.002)	(0.003)	(0.002)	(0.003)	(0.002)
Large city	0.008***	0.006***	0.008***	0.007***	0.008***	0.007***	0.008***	0.006***
	(0.002)	(0.002)	(0.002)	(0.002)	(0.002)	(0.002)	(0.002)	(0.002)
Country and survey wave dummies	Yes	Yes	Yes	Yes	Yes	Yes	Yes	Yes
Pseudo *R*^2^	0.092	0.142	0.092	0.146	0.091	0.144	0.092	0.144
Observations	142,468	142,468	121,607	121,607	126,803	126,803	126,680	126,680

Source: Authors’ estimation based on Gallup World Poll data

Notes: The table shows the average marginal effects from logistic regression estimates, whereby the dependent variable is having relatives abroad in models (1), (3), (5), and (7), and receiving remittances in models (2), (4), (6), and (8). Robust standard errors in parentheses. ****p* < 0.01, ***p* < 0.05, **p* < 0.1. The omitted categories are ages 15–35; completed primary education; married or living with partner; poorest 20%; no children in the household; small city/village; employed (full- or part-time, or self-employed); religion unimportant; no pain yesterday; satisfied with personal health; no health problem. Dummy variables for missing observations for each variable included but not reported. See Tables 11 and 12 in the Appendix for variable definitions and the list of countries included in each survey wave

## Footnotes

1 We discuss causality again in Section 2.3.

2 While our paper specifically examines international migration and receiving remittances from abroad, there is also an emerging literature on the well-being consequences of migrant remittances of rural-to-urban migrants and on the internal migrants themselves, for example in China (Akay et al. 2012, 2014a, b, 2016).

3 In this paper, we use the terms “affective well-being” and “hedonic well-being” synonymously.

4 We use the terms “life evaluations,” “evaluative well-being,” and “best possible life (BPL)” interchangeably.

5 We acknowledge a recent contribution by Hendriks et al. (2018) in the World Happiness Report, which appeared well after the original draft of this paper.

6 See Hendriks (2015, 2018) and Simpson (2013) for excellent summaries of the existing studies on happiness and migration.

7 While Gallup polls approximately 1000 respondents in each country, large countries such as China and Russia are oversampled and have at least 2000 respondents, while Puerto Rico has only 500. All respondents in the same country use the same interview method (either phone or face-to-face). Any bias stemming from the interview method (phone or face-to-face) on providing answers to emotional well-being questions is accounted for by country-fixed effects in the analysis. The phone sample design is based on random-digit dialing. The Kish grid or last birthday method is used to select one respondent within each household. For in-person interviews, Gallup uses a three-stage sampling procedure, whereby 100–135 household clusters per country are selected in the first stage (independent of previous-year samples). The second stage involves random route procedures to select sampled households. In the third stage, respondents are randomly selected within households using the Kish grid method, with only one respondent answering the questionnaire in each household. Gallup researchers re-weigh the data by adult household size to account for the lower probability of being in the sample for respondents in larger households. Gallup researchers also use post-stratification weights by age, gender and—where available—education and socio-economic status to ensure national representativeness. However, it is possible that the samples do not reflect the ethnic composition of the underlying populations, especially in ethnically diverse countries; given that Gallup does not report an ethnicity variable, we cannot check whether the national samples are representative of ethnic diversity.

8 While the Gallup World Poll started in 2005/6, remittance receipt, income, and employment status are only available starting in 2009. Moreover, the question on whether the respondent has family members abroad who left in the last 5 years is only available for 2007–2011. Therefore, the sample that contains all information we require for this analysis is 2009–2011.

9 We do not have data on the actual monetary value of either cash or in-kind remittances but rather only information on whether the respondent’s family receives them or not. We also recognize that respondents may underreport the receipt of remittances (although, arguably, respondents are less likely to underreport the receipt of remittances than the actual value of remittances). If, in addition, the underreporting of remittances receipt is related to country-level characteristics, such as inequality or weak institutions (because the respondents may worry that corrupt officials may be willing to get the data), caution should be applied when interpreting the country-group results (Section 3).

10 As a robustness check, we excluded the health variables from our control set, and the results remained unchanged (see Table 16 in the Appendix).

11 Note that the response distributions for these binary variables are typically similar to those for the longer scaled ordinal variables.

12 Nevertheless, even if such a panel dataset existed, it may have suffered from high attrition rates, thus making panel estimations unreliable.

13 We conducted additional analyses by the Human Development Index (HDI) group, which is another way of classifying countries according to their level of development. The results by HDI group—available on request or in the discussion paper version—are very similar to those by income group, especially for the evaluative well-being (BPL) estimations. The parallel is unsurprisingly given that per capita income is a major component of the HDI.

14 Another useful exercise, which we leave for future research, would be to check if less well-off people in poorer countries benefit from remittances more than their counterparts in richer countries—this could be because less well-off people in richer countries enjoy a better provision of public services and access to amenities.

15 Given our finding that remittances benefit people in less developed and more unequal countries, we further checked whether people from more deprived circumstances disproportionately benefit from remittances. Using education as a proxy for socio-economic status, we found that people with lower levels of education benefit most from remittances (Table 14). This corroborates our finding that remittances are associated with higher evaluative well-being in more deprived contexts.

16 This negative association could be due to the fact that the third quartile of the net migration rate indeed encompasses a range of very different countries—rich and poor, with positive and negative net immigration (from France, Germany, and Greece to Ecuador, Chad, and India)—and it is possible that the negative remittance coefficient reflects the fact that additional income from remittances affects BPL differently in these very different contexts.

17 We rely on the user-written command *ebalance* in Stata (Hainmueller and Xu 2013).

18 More precisely, entropy balancing is more efficient and reduces covariate imbalance compared with techniques such as propensity score matching (PSM). Unlike more traditional matching techniques such as PSM, which is usually implemented using an iterative trial and error process and requires researcher judgment regarding the tolerance level and the included covariates, entropy balancing achieves covariate balance by weighting the sample units. Entropy balancing also allows to balance on other moments of the covariate distribution such as the variance and kurtosis. In this paper, we balance on the mean and covariance of the covariates (see Table 15 for the balancing tests). Finally, unlike with PSM, in which some observations are dropped due to matching, the entropy balancing weights deviate as little as possible from base weights to prevent loss of information and maintain efficiency (Hainmueller 2012).

19 Results showing the determinants of having relatives abroad and remittance receipt are available in Table 18 in the Appendix.

20 A notable difference includes the results presented in model (8) whereby having a network of family and friends abroad on whom to rely on in times of need is negatively associated with depression reports, but receiving remittances is positively associated with depression. We leave the investigation of why it might be so for future research.

## References

[CR1] Abas MA, Punpuing S, Jirapramukpitak T, Guest P, Tangchonlatip K, Leese M, Prince M (2009). Rural–urban migration and depression in ageing family members left behind. Br J Psychiatry.

[CR2] Adams R (2011). Evaluating the economic impact of international remittances on developing countries using household surveys: a literature review. J Dev Stud.

[CR3] Adhikari R, Jampaklay A, Chamratrithirong A (2011). Impact of children’s migration on health and health care-seeking behavior of elderly left behind. BMC Public Health.

[CR4] Akay A, Constant A, Giulietti C, Guzi M (2017). Ethnic diversity and well-being. J Popul Econ.

[CR5] Akay A, Bargain O, Zimmermann KF (2017). Home sweet home? Macroeconomic conditions in home countries and the well-being of migrants. J Hum Resour.

[CR6] Akay A, Bargain OB, Giulietti C, Robalino JD, Zimmermann KF (2016). Remittances and relative concerns in rural China. China Econ Rev.

[CR7] Akay A, Constant A, Giulietti C (2014). The impact of immigration on the well-being of natives. J Econ Behav Organ.

[CR8] Akay A, Giulietti C, Robalino JD, Zimmermann KF (2014). Remittances and well-being among rural-to-urban migrants in China. Rev Econ Househ.

[CR9] Akay A, Bargain O, Zimmermann KF (2012). Relative concerns of rural-to-urban migrants in China. J Econ Behav Organ.

[CR10] Antman F (2010). Adult child migration and the health of elderly parents left behind in Mexico. Am Econ Rev.

[CR11] Antman FM (2012). Gender, educational attainment, and the impact of parental migration on children left behind. J Popul Econ.

[CR12] Beine M, Docquier F, Schiff M (2013). International migration, transfer of norms, and home country fertility. Can J Econ.

[CR13] Betz W, Simpson NB (2013). The effects of international migration on the well-being of native populations in Europe. IZA J Migr.

[CR14] Böhme MH, Persian R, Stöhr T (2015). Alone but better off? Adult child migration and health of elderly parents in Moldova. J Health Econ.

[CR15] Borraz F, Pozo S, Rossi M (2010). And what about the family back home? International migration and happiness in Cuenca, Ecuador. J Bus Strateg.

[CR16] Cárdenas M, Di Maro V, Sorkin I (2009). Migration and life satisfaction: evidence from Latin America. J Bus Strateg.

[CR17] Cai R, Esipova N, Oppenheimer M, Feng S (2014). International migration desires related to subjective well-being. IZA J Migr.

[CR18] Cattaneo C (2015). Opting in to opt out? Emigration and group participation in.

[CR19] Chauvet L, Mercier M (2014). Do return migrants transfer political norms to their origin country? Evidence from Mali. J Comp Econ.

[CR20] Cortes P (2015). The feminization of international migration and its effects on the children left behind: evidence from the Philippines. World Dev.

[CR21] De Neve J.-E., Diener E, Tay L, & Xuereb C (2013) The objective benefits of subjective well-being. In J. F. Helliwell, R. Layard, & J. Sachs (Eds.), *World Happiness Report* (pp. 54-79)

[CR22] Démurger S (2015) Migration and families left behind. *IZA World of Labor*

[CR23] Diener E, Chan MY (2011). Happy people live longer: subjective well-being contributes to health and longevity. Appl Psychol: Health Well-Being.

[CR24] Dreby J (2015). U.S. immigration policy and family separation: the consequences for children's well-being. Soc Sci Med.

[CR25] Exton C, Smith C & Vandendriessche D (2015) Comparing happiness across countries: does culture matter? *OECD working paper* no.62

[CR26] Ferrer-i-Carbonell A, Frijters P (2004). How important is methodology for the estimates of the determinants of happiness?. Econ J.

[CR27] Gallego JM, Mendola M (2013). Labour migration and social networks participation in southern Mozambique. Economica.

[CR28] Gallup Inc (2005). Gallup World Poll (2009–2015).

[CR29] Gartaula HN, Visser L, Niehof A (2012). Socio-cultural dispositions and wellbeing of the women left behind: a case of migrant households in Nepal. Soc Indic Res.

[CR30] Gibson J, McKenzie D, Stillman S (2011). The impacts of international migration on remaining household members: omnibus results from a migration lottery program. Rev Econ Stat.

[CR31] Graham C (2009). Happiness around the world: the paradox of happy peasants and miserable millionaires.

[CR32] Graham C, Higuera L, Lora E (2011). Which health conditions cause the most unhappiness?. Health Econ.

[CR33] Graham C, Nikolova M (2015). Bentham or Aristotle in the development process? An empirical investigation of capabilities and subjective well-being. World Dev.

[CR34] Graham C, Markowitz J (2011). Aspirations and happiness of potential Latin American immigrants. J Soc Res Policy.

[CR35] Graham E, Jordan LP (2011). Migrant parents and the psychological well-being of left-behind children in Southeast Asia. J Marriage Fam.

[CR36] Graham E, Jordan LP, Yeoh BS (2015). Parental migration and the mental health of those who stay behind to care for children in South-East Asia. Soc Sci Med.

[CR37] Goodman R (1997). The strengths and difficulties questionnaire: a research note. J Child Psychol Psychiatry.

[CR38] Hainmueller J (2012). Entropy balancing for causal effects: a multivariate reweighting method to produce balanced samples in observational studies. Polit Anal.

[CR39] Hainmueller J, Xu Y (2013). Ebalance: a Stata package for entropy balancing. J Stat Softw.

[CR40] Hendriks M (2015). The happiness of international migrants: a review of research findings. Migration Studies.

[CR41] Hendriks M (2018). Migrant happiness: insights into the broad well-being outcomes of migration and its determinants.

[CR42] Hendriks, M., Burger, M., Ray, J., & Esipova, N. (2018). Do international migrants increase their happiness and that of their families by migrating? In J. F. Helliwell, R. Layard, & J. Sachs (Eds.), *World Happiness Report 2018* (pp. 44-65)

[CR43] Howell RT, Kern ML, Lyubomirsky S (2007). Health benefits: Meta-analytically determining the impact of well-being on objective health outcomes. Health Psychol Rev.

[CR44] Ivlevs A (2015). Happy moves? Assessing the link between life satisfaction and emigration intentions. Kyklos.

[CR45] Ivlevs A, King R (2017). Emigration, remittances, and corruption experience of those staying behind. Public Choice.

[CR46] Ivlevs A, Veliziotis M (2018). Immigration and life satisfaction: the EU enlargement experience in England and Wales. Environ Plan A.

[CR47] Kroeger A, Anderson K (2014). Remittances and the human capital of children: new evidence from Kyrgyzstan during revolution and financial crisis. J Comp Econ.

[CR48] Levitt P (1998). Social remittances: migration driven local-level forms of cultural diffusion. Int Migr Rev.

[CR49] Longhi S (2014). Cultural diversity and subjective well-being. IZA J Migr.

[CR50] Marchetti-Mercer MC (2012). Those easily forgotten: the impact of emigration on those left behind. Fam Process.

[CR51] Mazzucato V, Cebotari V, Veale A, White A, Grassi M, Vivet J (2015). International parental migration and the psychological well-being of children in Ghana, Nigeria, and Angola. Soc Sci Med.

[CR52] Murphy M (2008). Variations in kinship networks across geographic and social space. Popul Dev Rev.

[CR53] Nikolova M, Roman M, Zimmermann KF (2017). Left behind but doing good? Civic engagement in two post-socialist countries. J Comp Econ.

[CR54] Nikolova M, Graham C (2015). In transit: the well-being of migrants from transition and post-transition countries. J Econ Behav Organ.

[CR55] Nobles J, Rubalcava L, Teruel G (2015). After spouses depart: emotional wellbeing among nonmigrant Mexican mothers. Soc Sci Med.

[CR56] O'Donnell G (2013) Using well-being as a guide to policy. In J. Helliwell, R. Layard, & J. Sachs (Eds.), *World Happiness Report* (pp. 98-111)

[CR57] OECD (2011). How's life?: measuring well-being.

[CR58] OECD (2013). OECD guidelines on measuring subjective well-being.

[CR59] Office for National Statistics (2013). Personal well-being in the UK, 2012/13.

[CR60] Oswald A, Proto E, Sgroi D (2015). Happiness and productivity. J Labor Econ.

[CR61] Otrachshenko V, Popova O (2014). Life (dis)satisfaction and the intention to migrate: evidence from Central and Eastern Europe. J Socio-Econ.

[CR62] Sabatini F (2014). The relationship between happiness and health: evidence from Italy. Soc Sci Med.

[CR63] Scheffel J, & Zhang Y (2015) To what extent does rural migration affect the elderly “left-behind”? A paper presented at the 4th SOLE/EALE world meetings, Montreal

[CR64] Simpson N (2013) Happiness and migration. In K. F. Zimmermann & a. F. Constant (Eds.), international handbook on the economics of migration (pp. 393-407): Edward Elgar Publishing Limited

[CR65] Skeldon R (2008). International migration as a tool in development policy: a passing phase?. Popul Dev Rev.

[CR66] Stone AA, & Mackie C (2014) *Subjective Well-Being: Measuring Happiness, Suffering, and Other Dimensions of Experience*: National Academies Press 24432436

[CR67] Taylor EJ (1999). The new economics of labour migration and the role of remittances in the migration process. Int Migr.

[CR68] UNDP (2009). Overcoming barriers: human mobility and development.

[CR69] Vanore M, Mazzucato V, Siegel M (2015). ‘Left behind’ but not left alone: parental migration & the psychosocial health of children in Moldova. Soc Sci Med.

[CR70] Waidler J, Vanore M, Gassmann F, Siegel M (2016) Does it matter where the children are? The wellbeing of elderly people ‘left behind’by migrant children in Moldova. Ageing Soc:1–26

[CR71] Wen M, Su S, Li X, Lin D (2015). Positive youth development in rural China: the role of parental migration. Soc Sci Med.

[CR72] Wu Q, Lu D, Kang M (2015). Social capital and the mental health of children in rural China with different experiences of parental migration. Soc Sci Med.

[CR73] Xie J-f, Ding S-g, Zhong Z-q, Yi Q-f, Zeng S-n, Hu J-h (2014). Mental health is the most important factor influencing quality of life in elderly left behind when families migrate out of rural China. Revista Latino-Americana de Enfermagem.

[CR74] Yang D (2008). International migration, remittances and household investment: evidence from Philippine migrants’ exchange rate shocks. Econ J.

